# The Crosstalk of Pathways Involved in Immune Response Maybe the Shared Molecular Basis of Rheumatoid Arthritis and Type 2 Diabetes

**DOI:** 10.1371/journal.pone.0134990

**Published:** 2015-08-07

**Authors:** Xuyan Niu, Cheng Lu, Cheng Xiao, Na Ge, Miao Jiang, Li Li, Yanqin Bian, Gang Xu, Zhaoxiang Bian, Ge Zhang, Aiping Lu

**Affiliations:** 1 Institute of Basic Research in Clinical Medicine, China Academy of Chinese Medical Sciences, Beijing, China; 2 China-Japan Friendship Hospital, Beijing, China; 3 Institute for Advancing Translational Medicine in Bone & Joint Diseases,School of Chinese Medicine, Hong Kong Baptist University, Hong Kong, China; 4 E-Institute of Chinese Traditional Internal Medicine, Shanghai Municipal Education Commission, Shanghai, China; Yale University, UNITED STATES

## Abstract

Rheumatoid arthritis (RA) and Type 2 diabetes (T2D) are both systemic diseases linked with altered immune response, moderate mortality when present together. The treatment for both RA and T2D are not satisfied, partly because of the linkage between them has not yet been appreciated. A comprehensive study for the potential associations between the two disorders is needed. In this study, we used RNA sequencing to explore the differently expressed genes (DEGs) in peripheral blood mononuclear cells (PBMC) of 10 RA and 10 T2D patients comparing with 10 healthy volunteers (control). We used bioinformatics analysis and the Ingenuity Pathways Analysis (IPA) to predict the commonalities on signaling pathways and molecular networks between those two diseases. 212 DEGs in RA and 114 DEGs in T2D patients were identified compared with healthy controls, respectively. 32 DEGs were shared between the two comparisons. The top 10 shared pathways interacted in cross-talking networks, regulated by 5 shared predicted upstream regulators, leading to the activated immune response were explored, which was considered as partly of the association mechanism of this two disorders. These discoveries would be considered as new understanding on the associations between RA and T2D, and provide novel treatment or prevention strategy.

## Introduction

Rheumatoid arthritis (RA) and Type 2 diabetes (T2D) are both complex and chronic systemic diseases, with increased risk of cardiovascular disease (CVD), as important causes of morbidity and mortality, especially when accompanied with each other [1, 2]. As known, RA is an autoimmune disease characterized by inflammation in multiple joints, with progressive and chronic activation of the immune system [3, 4]. T2D is a metabolic disorder characterized by defects in glucose uptake in response to insulin [5]. Although T2D is not considered as autoimmune disease traditionally, its state is linked with altered immune response including low-graded inflammation had been revealed [6]. In addition, several reports confirmed that patients with RA had characteristics placing them at high risk for T2D [7, 8]. However, the correlation between RA and T2D cannot been ruled out, and whether the association is related with immune response has not yet been illuminated, especially in the lack of comprehensive study to look at the molecular association and commonly shared mechanisms between these two disorders [9].

The disturbance of signaling pathways on inflammation and immune response was considered as one of important reasons for both RA and T2D respectively [6, 10]. Evidences implicated that the pro-inflammatory cytokines TNF-α and IL-6, which were key mediators of inflammation in RA, were overproduced in visceral adipose tissue and impaired insulin receptor signaling to cause insulin resistance (IR) in T2D [11, 12]. Other inflammatory cytokines, such as elevated levels of IL-2 in both serum and synovial tissues were appeared to be associated with both RA and T2D, and correlated with insulin sensitivity in patients with RA [13, 14]. However, the complex interactions among cytokines in RA and T2D were yet to be fully elucidated.

There are independent studies showing that drug developed for treatment of RA can also be used to treat T2D. Recent studies have demonstrated that treatment with TNF antagonists alters the lipid profile and improves insulin sensitivity in patients with RA, but not so effective in early stage [10, 15]. Another example, the thiazolidinediones (TZD) is used in the treatment of T2D, as wells as show the inhibition of T-cell activation and inflammatory disease [16]. These classes of drugs are of growing importance as a therapeutical approach in inflammatory and autoimmune diseases such as RA by regulating IL-17A, IL-22, and IFN-γ levels, but with a few significant cardiovascular side effects [17, 18]. Thus, because of the limited targets and side effects of those drugs, as well as the complexity of both the two disorders, the discovery of satisfied and novel treatment or drugs targets effective on both RA and T2D was blocked no doubt [10, 19]. So that, the commonality of RA and T2D at molecular level mechanism screening in order to provide more information for developing conjunct treatment targets on both RA and T2D maybe one strategy was called.

Recently, several high-throughput techniques are used to study the expression of mRNAs, such as the next-generation sequencing (NGS) platforms, which have the advantages of greater sensitivity and more precise quantification, providing a more complete result of the transcriptome in studies of gene expression compared with a microarray [20–22]. Measurements of mRNA expression by RNA sequencing have proven to be valuable for identification of the molecular changes that occur in cells, provide clues for molecular networks in diseases process [23]. Currently, one of NGS protocols: 3′- tag digital gene expression (DGE) developed by Illumina (Illumina Inc., San Diego, CA) have been widely used for transcriptome studies [24]. There are reports focusing on the molecular mechanisms of pathophysiologic changes during RA or T2D independently by transcriptome or gene expression profiles technology [23, 25], but few reports concerning the associations between RA and T2D at the transcriptome level, neither in-depth study on the mechanisms and the molecular networks on the commonly shared pathways between RA and T2D [26].

In the current study, we applied the RNA sequencing technology for the peripheral blood mononuclear cells (PBMC) RNA of RA and T2D patients to study the gene expression changes and identify the differentially expressed genes (DEGs) by comparison with healthy volunteers. Furthermore, we performed bioinformatics analysis and network analysis to identify the associated pathways of RA and T2D. We predicted upstream regulators related to the identified genes using Ingenuity Pathways Analysis (IPA, http://www.ingenuity.com) software, which enables the discovery visualization and exploration of molecular interaction networks [27]. The purpose of this study is to get a comprehensive understanding of the associations of RA and T2D by identification of the canonical pathways, biological network mechanisms, upstream regulators based on DEGs by DGE, which may provide new insights on the pathphysiology and new drug targets for these two diseases.

## Methods

### Patients

RA, T2D patients and healthy volunteers were recruited at China-Japan Friendship Hospital in Beijing. The active RA sufferers were selected via their general practitioner from the outpatient rheumatology clinic as defined by the 1987 American College of Rheumatology revised criteria and 2010 American College of Rheumatology/ European League Against Rheumatism classification criteria for RA [28, 29]. Because RA predominates in females with two to three times more common, we choose female patients as observed subjects [30] in this study. T2D patients were defined by the American Diabetes Association (ADA) 1997 diagnostic criteria [31].

Exclusion criteria included current or prior treatment with prednisone, systolic blood pressure < 90 or > 150 mm Hg, heart rate < 50 or > 130 bpm, hyperlipdemia, dyslipidemia, history of smoking. Patients with severe diseases lung, liver, kidney, and those with mental or blood system disturbances, as well as women who were pregnant, breast-feeding or planning to have pregnancy in the next 8 months were also excluded from the study. For RA patients, the patients who continuously received non-steroidal anti-inflammatory drugs、DMARDs、biological agents or corticosteroids for over 6 months or those who received the aforementioned medicines within one month were excluded from the study. As for T2D patients, the exclusion criteria were serious complications of diabetes and exercise contraindications by electrocardiogram, urinalysis, ophthalmology, or blood pressure checks; severe myocardial ischemia, diabetic nephropathy with renal failure, positive urine ketone bodies, proliferative retinopathy, or severe hypertension; fasting blood glucose exceeding 16.7 mmol/L.Of course, the RA patients included were not with any type of diabetes mellitus (DM), and the T2D patients included were not with RA either. The persons in control group were considered healthy if their medical history did not reveal any chronic diseases, endemic infectious diseases or autoimmune diseases, and their physical examination and blood tests failed to show otherwise.

The general information of RA, T2D patients and healthy controls was shown in **Table 1**. Blood samples were collected from 10 female RA patients, 10 female T2D patients. 10 healthy age- matched female were selected as healthy controls for blood collection. For each individual participated in this study, 5 mL of peripheral blood samples were collected in the morning before breakfast. The PBMC were prepared immediately, and then kept in Trizol under -80°C until analysis. All protocols involving human subjects had been approved by the ethics committee of China-Japan Friendship Hospital (ethics ID: 2014–58), and informed consents were signed by all participants before commencing the study.

**Table 1:** General information of RA, T2D patients and the healthy control.

Characteristic	Control	RA	T2D
**Female Cases**	10	10	10
**Age, Mean years ± SD**	45.8±3.5	54.5±7.1	50.3±4.9
**Disease duration, Mean years ± SD**	/	2.1±1.1	2.9±1.3
**BMI (kg/m** ^**2**^ **), mean ± SD**	22.34±1.83	25.81±2.62	25.60±3.45
**ESR (mm/h), mean ± SD**	/	39.14±29.53	/
**CRP (mg/L), mean ± SD**	/	15.48±15.40	/
**RF (IU/mL), mean ± SD**	/	63.76±71.81	/
**WBC×10** ^**9**^ **/L, mean ± SD**	4.92±1.10	6.09±1.50	6.57±1.70
**HGB (g/L), mean ± SD**	126.2±13.89	122.38±14.74	130.00±12.85
**PLT×10** ^**9**^ **/L, mean ± SD**	228.7±28.69	246.75±75.06	246.60±66.54
**HbA1c %, mean ± SD**	/	/	7.9±1.3
**Total Cholesterol mmol/L, mean ± SD**	/	/	4.78±0.94
**HDL Cholesterol mmol/L, mean ± SD**	/	/	1.22±0.36
**LDL- Cholesterol mmol/L, mean ± SD**	/	/	2.38±0.61
**Triacylglycerol, mmol/L, mean ± SD**	/	/	1.56±0.91
**Glucose mmol/L, mean ± SD**	/	/	7.53±2.3
**Insulin mU/L, mean ± SD**	/	/	15.2±11.3
**Leptin μg/L, mean ± SD**	/	/	8.38±6.1

(BMI: Body Mass Index, ESR: Erythrocyte Sedimentation Rate, CRP: C-reactive protein, RF: Rheumatoid Factor, WBC: White Blood Cell, HGB: hemoglobin, PLT: Platelets)

### PBMC isolation and total RNA extraction

PBMC were isolated by density gradient centrifugation over Ficoll-Hypaque gradient solution according to the manufacturer’s instructions. Heparinized whole blood 5mL was diluted to 10mL with phosphate buffered saline (PBS, pH 7.4), layered on top of 5mL Ficoll-Hypaque and centrifuged for 30mins at 400×g. PBMC were aspirated, washed twice, suspended in PBS and counted with a hemocytometer. PBMCs was lysed in Trizol reagent at a ratio of 1 mL Trizol per 1 × 10^7^ cells and stored at −80°C until further processing.

PBMC samples from RA or T2D patients and healthy controls were selected for total RNA isolation. Total RNA was isolated by Trizol extraction method according to the manufacturer’s protocol. Total RNA was quantified with a NanoDrop ND-1000 spectrophotometer followed by quality assessment with the 2100 Bioanalyzer using an RNA 6000 LabChip kit according to the manufacturer’s instructions. Acceptable quality values were in the 1.8–2.2 range for A260/A280 ratios, and for each total RNA sample, the RNA Integrity Number was >7.0.

### DGE tag profiling and identification of differentially expressed genes

For each sample, 4 ug of total RNA were purified by adsorption of biotin oligo magnetic beads. After mRNA’s binding, cDNA synthesis was performed. Double strand cDNA was introduced into cDNA fragment digested by *NlaIII* endonuclease and these binging fragment containing sequences of CATG site and adjacent poly A tail in 3’ end. After the precipitation of 3’ cDNA fragment, Illumina adaptor 1 was added to 5’ end. Both adaptor 1 and CATG site can be recognized by *MmeI*, which cut at downstream CATG site and produce fragment of 17 bp tags with adaptor 1. Adaptor 2 was added to the 3’ end of these tags after getting rid of fragment with beads in 3’ end. Then these sequences were prepared for Solexa sequencing (GEO ID: SRA276834).

Clean-tags were obtained by filtering the adaptor sequences and removing low-quality sequences (containing ambiguous bases). Only the tags with perfect match or one mismatch were further considered and annotated based on the reference genes. The expression level of each gene was estimated by the frequency of clean tags and then normalized to TPM (number of transcripts per million clean tags) [24], which is a standard method and extensively used in DGE analysis [32]. TPM indicates reads per kilobase of transcript per million of sequenced reads. The expression level of each gene was measured by thenormalized number of matched clean tags.

The number of tags mapped to a given gene was considered to represent the expression level of this gene. Expression levels of a gene from two distinct samples were compared to calculate an expression difference. We identified the DEGs in RA *vs*. control (we define as ‘RA’ following) and T2D *vs*. control (we define as ‘T2D’ following) groups. Significance values for expression differenceswere determined using a modified exact test, similar to Fisher’s exact test. The *P*-values were adjusted using the Benjamini and Hochberg false discovery rate (FDR) of 1%. We classified the gene as differentially expressed only when the expression difference was more than 1.5 folds with adjusted *p*-value <0.01.

### Pathway and network analysis by IPA

The Gene Bank accession number and matched identified DEGs in RA and T2D groups were set up as the identifiers of two datasets. Each dataset was uploaded into the Ingenuity Pathways Analysis system (IPA, http://www.ingenuity.com) that enabled the discovery visualization and exploration of molecular interaction in order to identify the biological mechanisms, pathways and functions most relevant to the experimental datasets, genes, or proteins of interest. The proof-of-knowledge based IPA was performed to characterize the biomarkers confirmed by the pattern recognition analyses, so as to evaluate the biomarkers based on their metabolic or signaling associations in canonical pathways and biological networks [33]. We utilized IPA analysis system with the ‘Core analysis’ module to analyze the identified DEGs of RA and T2D groups; compare the canonical pathways and biological networks between RA and T2D groups by ‘Comparison’ module of IPA in the meantime. In this study, the score is -10 logarithms of Fisher’s exact test *p*-values in canonical pathway analysis by IPA. Significances for biological functions were then assigned to each network by determining a *p*-value for the enrichment of the genes in the network for such functions compared with the whole Ingenuity Pathway Knowledge Base as a reference set.

### Qualitative real time polymerase chain reaction (Q-PCR)

The Q-PCR method was used to validate the top shared 12 candidate genes: C4BPA, ARG1, MMP9, DEFA1, CYP4F3, LTF, MPO, DEFA4, MMP8, RBP1, SNAI and MT2A. Briefly[34], the forward and reverse primers were designed using Primer Express Software. Five hundred ng of total RNA from each sample was used as a template to synthesize cDNA according to the iScript cDNA synthesis kit manufacturer's instructions. The qPCR was conducted using Power SYBR Green PCR Master Mix on 7900HT Fast Real-Time PCR System. Four biological samples for each time point of infection and mock infection were used in the qRT-PCR, and each was conducted in duplicate. The relatihttps://www.youtube.com/watch?v=M-elI3cO4x4ve transcript level of each gene was calculated according to the 2^−Ct^, for unnormalized genes, and the 2^−ΔΔCt^ method, for the genes normalized to 28S. The relative transcript levels in the healthy control was considered to be 1 and the relative transcript levels at RA and T2D group was calculated accordingly. A Student's t-test was performed on the data.

## Results

### Identified DEGs in RA and T2D patients compared with control

**Fig 1:**
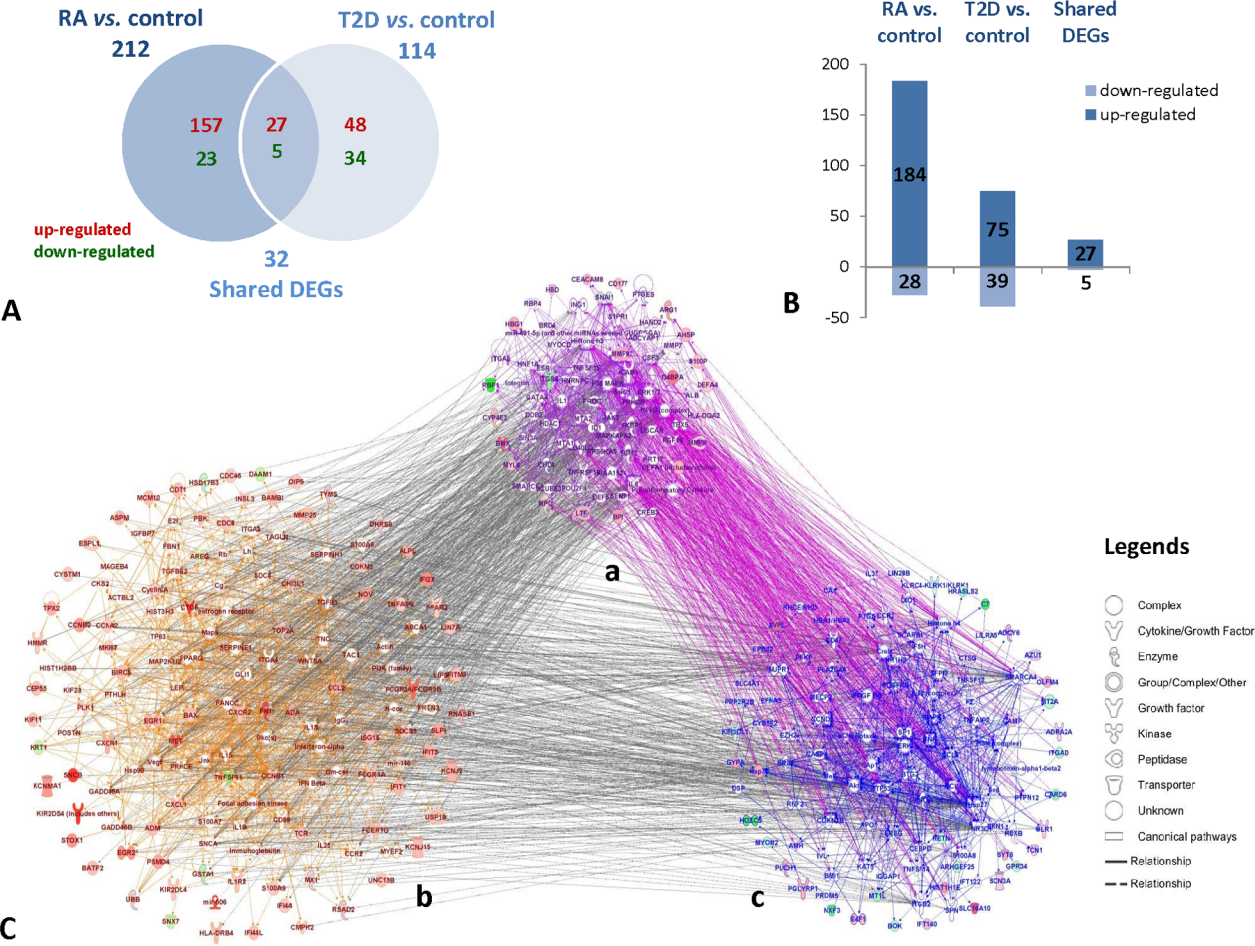
The number of DEGs in RA *vs*. control and T2D *vs*. control. **A**: Venn diagram was showing number of uniquely up-regulated (red) or down-regulated (green) genes comparing RA, T2D to control and shared DEGs; **B**: bar diagram was indicating number of DEGs in groups of RA *vs*. control, T2D *vs*. control and shared DEGs between them; **C**: the molecular networks of relationships between RA and T2D based on the DEGs; **Ca**: the shared molecular networks of RA and T2D based on the shared DEGs between RA *vs*. control and T2D *vs*. control, with nodes colored by **violet**; **Cb**: the molecular networks of RA based on the DEGs in RA *vs*. control, with nodes colored by **orange**; **Cc**: the molecular networks of T2D based on the DEGs in T2D *vs*. control, with nodes colored by **blue**.

By DGE technique, we identified genes differentially expressed in RA compared with control (RA *vs*. control) and T2D compared with control (T2D *vs*. control), we compared pairs of DGE profiles of RA *vs*. control and T2D *vs*. control further. 212 genes in RA *vs*. control and 114 genes in T2D *vs*. control were identified differentially expressed, as shown in Table A in S1 File, Table B in S2 File, Fig 1A and 1B. There were 184 up-regulated and 28 down-regulated DEGs found in RA *vs*. control; while 75 up-regulated and 39 down-regulated DEGs found in T2D *vs*. control. There were 32 DEGs shared in both RA *vs*. control and T2D *vs*. control, as shown in Fig 1A, 1B and Table C in S3 File.

### The shared molecular networks of relationships between RA and T2D based on the DEGs

Based on the DEGs, we built the molecular network of RA *vs*. control and T2D *vs*. control respectively by IPA software, as shown in Fig 1Cb and 1Cc. In order to find the associations between the two group DEGs, we further built the molecular network based on the shared 32 DEGs between RA *vs*. control and T2D *vs*. control, which was the network Ca in Fig 1. Overlapping the molecular networks of DEGs in the RA, T2D and shared DGEs between the two groups, so many interacted links based on DEGs between them were found among network Ca, Cb and Cc in Fig 1, which reminded us the complex association of RA and T2D were exist at transcriptome level.

### The shared signaling pathways and bio-function between RA and T2D

**Fig 2:**
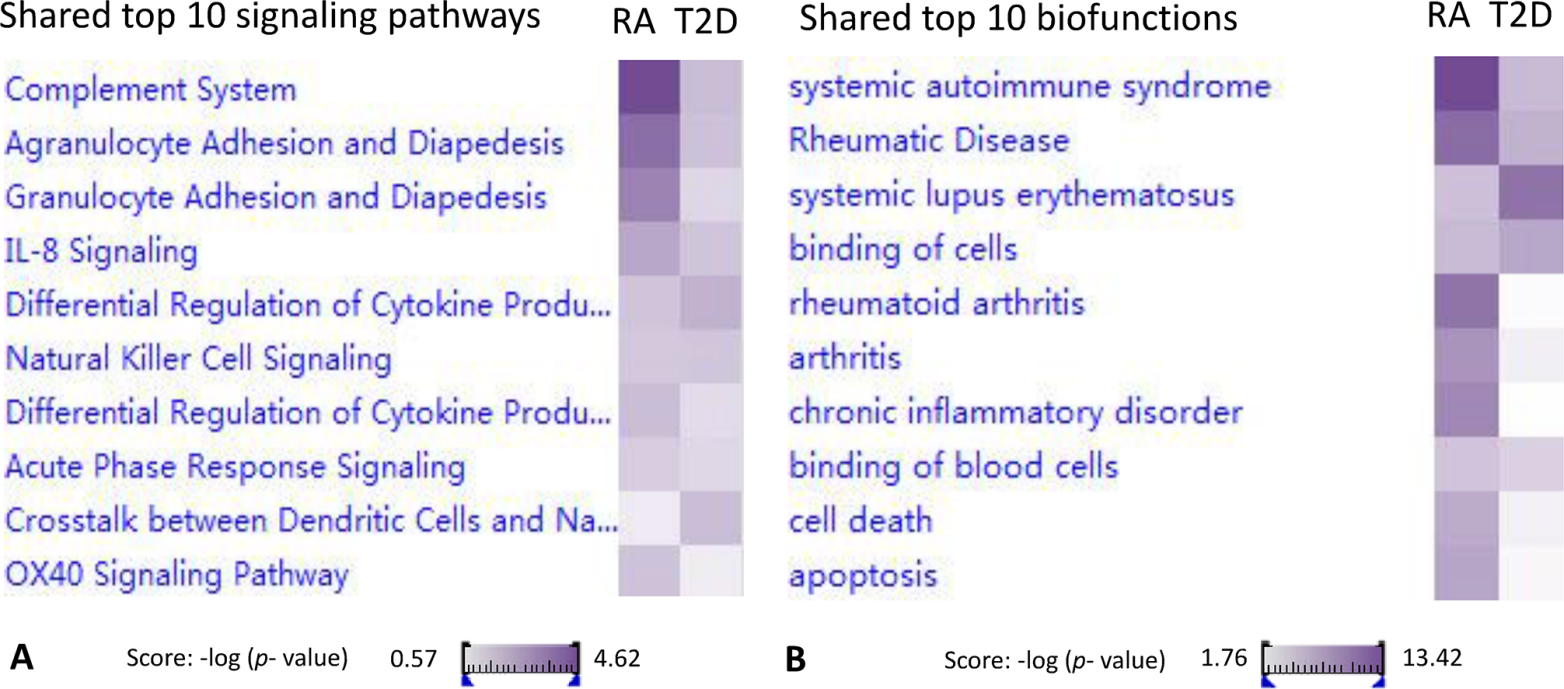
Heat-map of top 10 commonly shared signaling pathways and bio-functions between RA and T2D. The heat map of shared signaling and bio-functions were made in the comparison analysis platform by IPA software. The depth of the color represented the score (-log (*p*-value)) of each pathway or bio-function in different groups. A: The shared top 10 canonical signaling pathways between RA and T2D; B: The shared top 10 bio-functions between RA and T2D.

**Fig 3:**
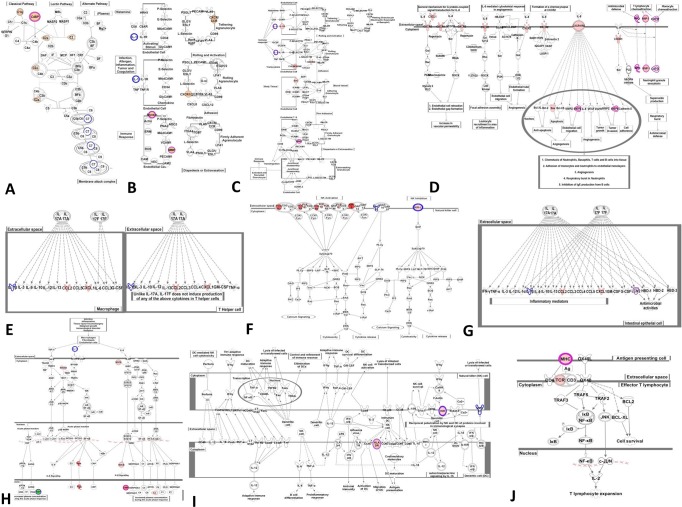
The commonly shared top 10 signaling pathways between RA and T2D in details. **A**: completement system; **B**: agranulocyte adhesion and diapedesis; **C**: granulocyte adhesion and diapedesis; **D**: IL-8 signaling; **E**: differential regulation of cytokine production in macrophages and T helper cells by IL-17A and IL-17F; **F**: Natural killer cell signaling; **G**: differential regulation of cytokine production in intestinal epithelial cells by IL-17A and IL-17F; **H**: acute phase response signaling; I: crosstalk between dendritic cells and natural killer cells; **J**: OX40 signaling pathways. **Red nodes**: up-regulated DEGs in RA *vs*. control group; green nodes: down-regulated DEGs in RA *vs*. control group; **violet circles**: up-regulated DEGs in T2D *vs*. control group; **blue circles**: down-regulated DEGs in T2D *vs*. control group.

Top 10 shared signaling pathways found in both RA and T2D groups, chosen based on score (-log (*p*-value)) of each pathway (Figs 2A, 3 and Table 2) were: completement system, agranulocyte adhesion and diapedesis, granulocyte adhesion and diapedesis, IL-8 signaling, differential regulation of cytokine production in macrophages and T helper cells by IL-17A and IL-17F, Natural killer cell signaling, differential regulation of cytokine production in intestinal epithelial cells by IL-17A and IL-17F, acute phase response signaling, crosstalk between dendritic cells and natural killer cells, and OX40 signaling pathways. The categories of these signaling pathways were concentrated on cellular immune response, cytokine signaling, and humoral immune pathways. Among those pathways, there were 5 canonical pathways with the scores (-log (*p*-value)) higher than 1.3 (*p*-value < 0.05) in both RA and T2D groups. These 5 pathways were considered more important: complement system, agranulocyte adhesion and diapedesis, IL-8 signaling, Natural killer cell signaling, and differential regulation of cytokine production in intestinal epithelial cells by IL-17A and IL-17F. There were 21 DEGs shared in both the RA and T2D groups, which were also involved in the 10 shared pathways: CYP4F3↑, DEFA4↑, DEFA1↑, MMP8↑, MMP9↑, MT2A↓, ITGB4↓, RBP1↓, SNAI1↓, ARG1↑, MPO↑, LTF↑, C4BPA↑, MYL4↑, HLA-DQA2↑, CA1↑, SELENBP1↑, BPI↑, CD177↑, CA4↑, and BMX↑. The detailedshared signaling pathways and DEGs involved in were shown in Fig 3.

**Table 2:** The top 10 shared signaling pathways and bio-functions between RA and T2D and DEGs involved in (the bold and italic words represents the same DEGs shared between RA and T2D) (Score means–log (*p*-value)).

NO.No.	Name	Category	Score in RA group	Score in T2D group	DEGs of RA group involved in	DEGs of T2D group involved in
***The top 10 shared canonical pathways*:**						
**1**	**Complement system**	Humoral immune response	4.57	1.79	C2***↑***, C1QA***↑***, C1QB***↑***, C1QC***↑***, ***C4BPA↑***	C7↓, ***C4BPA↑***
**2**	**Agranulocyte adhesion and diapedesis**	Cellular immune response	3.77	1.65	***MMP8↑***, ***MMP9↑***, ***MYL4↑***, CCL2***↑***, CXCL1***↑***, CXCR1***↑***, CXCR2***↑***, FN1***↑***, MMP25***↑***	***MMP8↑***, ***MMP9*** *↑*, ***MYL4***↑, IL1β↓
**3**	**Granulocyte adhesion and diapedesis**	Cellular immune response	3.24	1.10	***MMP8↑***, ***MMP9↑***, CCL2***↑***, CXCL1***↑***, CXCR2***↑***, MMP25***↑***, IL1R2***↑***, SDC3***↑***	***MMP8↑***, ***MMP9↑***, IL1β↓
**4**	**IL-8 signaling**	Cellular immune response	2.32	1.59	BAX↑, CXCR1↑, CXCL1↑, CXCR2↑, ***DEFA1↑*, *MMP9↑***, ***MPO↑***	AZU1↑, ***DEFA1*↑**, ***MMP9↑***, ***MPO↑***
**5**	**Differential regulation of cytokine production in macrophages and T helper cells by IL-17A and IL-17F**	Cytokine signaling	1.77	0.98	CCL2***↑***, CXCL1***↑***	IL1β↓
**6**	**Natural killer cell signaling**	Cellular immune response	1.47	1.55	FCER1G***↑***, FCGR3B***↑*,** KIR2DL4***↑***, KIR2DS4***↑***	KIR2DL5B↓, KIR3DL1↓, KLRK1↓
**7**	**Differential regulation of cytokine production in intestinal epithelial cells by IL-17A and IL-17F**	Cytokine signaling	1.56	2.07	CCL2***↑***, CXCL1***↑***	IL1β↓, LCN2***↑***
**8**	**Acute phase response signaling**	Cytokine signaling	1.19	0.63	C2***↑***, ***C4BPA↑***, FN1***↑***, ***RBP1***↓, SOCS3***↑***	***C4BPA↑***, ***RBP1***↓, IL1β↓
**9**	**Crosstalk between dendritic cells and natural killer cells**	Cellular immune response	0.58	1.77	HLA-DRB4↑, KIR2DL4↑	KIR2DL5B↓, KIR3DL1↓, KLRK1↓
**10**	**OX40 signaling pathways**	Cellular immune response	1.64	0.55	FCER1G***↑***, ***HLA-DQA2↑***, HLA-DRB4***↑***	***HLA-DQA2↑***
***The top 10 shared biofunctions*:**						
**1**	**Systemic autoimmune syndrome**	Diseases and disorder	13.27	6.33	***ARG1↑***, ***BPI↑***, ***CA1↑***, ***CYP4F3↑***, ***DEFA4↑***, ***DEFA1↑***, ***HLA-DQA2↑***, ***LTF↑***, ***MMP8↑***, ***MMP9↑***, ***MPO↑*** and other 33 molecules.	***ARG1↑***, ***BPI↑***, ***CA1↑***, ***CYP4F3↑***, ***DEFA4↑***, ***DEFA1↑***, ***HLA-DQA2↑***, ***LTF↑***, ***MMP8↑***, ***MMP9↑***, ***MPO↑*** and other 11 molecules.
**2**	**Rheumatic disease**	Diseases and disorder	11.34	6.90	***ARG1↑***, ***BPI↑***, ***CYP4F3↑***, ***DEFA4↑***, ***DEFA1↑***, ***MPO↑***, ***MMP8↑***, ***MMP9↑***, ***HLA-DQA2↑***, ***LTF↑***, ***SELENBP1↑*** and other 32 molecules.	***ARG1↑***, ***BPI↑***, ***CYP4F3↑***, ***DEFA4↑***, ***DEFA1↑***, ***MPO↑***, ***MMP8↑***, ***MMP9↑***, ***HLA-DQA2↑***, ***LTF↑***, ***SELENBP1↑*** and other 13 molecules.
**3**	**System lupus erythematosus**	Diseases and disorder	5.88	10.94	***BPI↑***, ***DEFA4↑***, ***MPO↑***, ***MMP8↑***, ***MMP9↑***, ***LTF↑***, and other 4 molecules.	***BPI↑***, ***DEFA4↑***, ***MPO↑***, ***MMP8↑***, ***MMP9↑***, ***LTF↑***, and other 6 molecules.
**4**	**Binding of cells**	Molecular and cellular functions	4.31	7.56	***BPI↑***, ***CD177↑***, ***DEFA1↑***, ***LTF↑***, ***ITGB4***↓, ***SNAI1***↓ and other 11 molecules.	***BPI↑***, ***CD177↑***, ***DEFA1↑***, ***LTF↑***, ***ITGB4***↓, ***SNAI1***↓and other 8 molecules.
**5**	**Rheumatic arthritis**	Diseases and disorder	10.89	1.90	***ARG1↑***, ***CYP4F3↑***, ***DEFA1↑***, ***HLA-DQA2↑***, ***LTF↑***, ***MMP9↑***, ***MPO↑***, and other 28 molecules.	***ARG1↑***, ***CYP4F3↑***, ***DEFA1↑***, ***HLA-DQA2↑***, ***LTF↑***, ***MMP9↑***, ***MPO↑***, and other 4 molecules.
**6**	**arthritis**	Diseases and disorder	8.84	2.80	***ARG1↑***, ***CYP4F3↑***, ***DEFA1↑***, ***HLA-DQA2↑***, ***LTF↑***, ***MMP9↑***, ***MPO↑***, ***SELENBP1↑*** and other 28 molecules.	***ARG1↑***, ***CYP4F3↑***, ***DEFA1↑***, ***HLA-DQA2↑***, ***LTF↑***, ***MMP9↑***, ***MPO↑***, ***SELENBP1↑***and other 7 molecules.
**7**	**Chronic inflammatory disorder**	Diseases and disorder	9.61	1.72	***ARG1↑***, ***CYP4F3↑***, ***DEFA1↑***, ***HLA-DQA2↑***, ***LTF↑***, ***MMP9↑***, ***MPO↑***, and other 32 molecules.	***ARG1↑***, ***CYP4F3↑***, ***DEFA1↑***, ***HLA-DQA2↑***, ***LTF↑***, ***MMP9↑***, ***MPO↑***, and other 6 molecules.
**8**	**Binding of blood cells**	Molecular and cellular functions	5.66	4.83	***BPI***↑, ***LTF***↑, and other 8 molecules.	***BPI***↑, ***LTF***↑, and other 5 molecules.
**9**	**Cell death**	Molecular and cellular functions	7.37	2.67	***ARG1↑***, ***BMX↑***, ***BPI***↑, ***CA4***↑, ***CD177↑***, ***DEFA1↑***, ***ITGB4***↓, ***LTF***↑, ***MMP9↑***, ***MPO↑***,***MT2A***↑, ***SNAI1***↓ and other 47 molecules.	***ARG1↑***, ***BMX↑***, ***BPI***↑, ***CA4***↑, ***CD177↑***, ***DEFA1↑***, ***ITGB4***↓, ***LTF***↑, ***MMP9↑***, ***MPO↑***,***MT2A***↑, ***SNAI1***↓ and other 15 molecules.
**10**	**Apoptosis**	Molecular and cellular functions	7.67	2.30	***ARG1↑***, ***BMX↑***, ***BPI***↑, ***CA4***↑, ***ITGB4***↓, ***LTF***↑, ***MMP9↑***, ***MPO↑***, ***MT2A***↑, ***SNAI1***↓ and other 41 molecules.	***ARG1↑***, ***BMX↑***, ***BPI***↑, ***CA4***↑, ***ITGB4***↓, ***LTF***↑, ***MMP9↑***, ***MPO↑***, ***MT2A***↑, ***SNAI1***↓ and other 12 molecules.

Further, the top 10 biofunctions shared in both the RA and the T2D groups found based on score (-log (*p*-value)) of each biofunction (Fig 2B and Table 2) were: systemic autoimmune syndrome, rheumatic disease, system lupus erythematosus, binding of cells, rheumatic arthritis, arthritis, chronic inflammatory disorder, binding of blood cells, cell death and apoptosis. Most of those functions were related with immune disoder, although concentrated on two conventional categories of biofunctions: molecular and cellular functions, and diseases and disorder. There were 19 key DEGs found commonly shared in both the RA and the T2D groups associated with these shared bio-functions: ARG1↑, BPI↑, CA1↑, CYP4F3↑, DEFA4↑, DEFA1↑, HLA-DQA2↑, LTF↑, MMP8↑, MMP9↑, MPO↑, SELENBP1↑, BPI↑, CD177↑, ITGB4↓, SNAI1↓, MT2A↑, BMX↑, CA4↑.

### The shared upstream regulators network of DEGs in RA and T2D predicted by IPA

**Fig 4:**
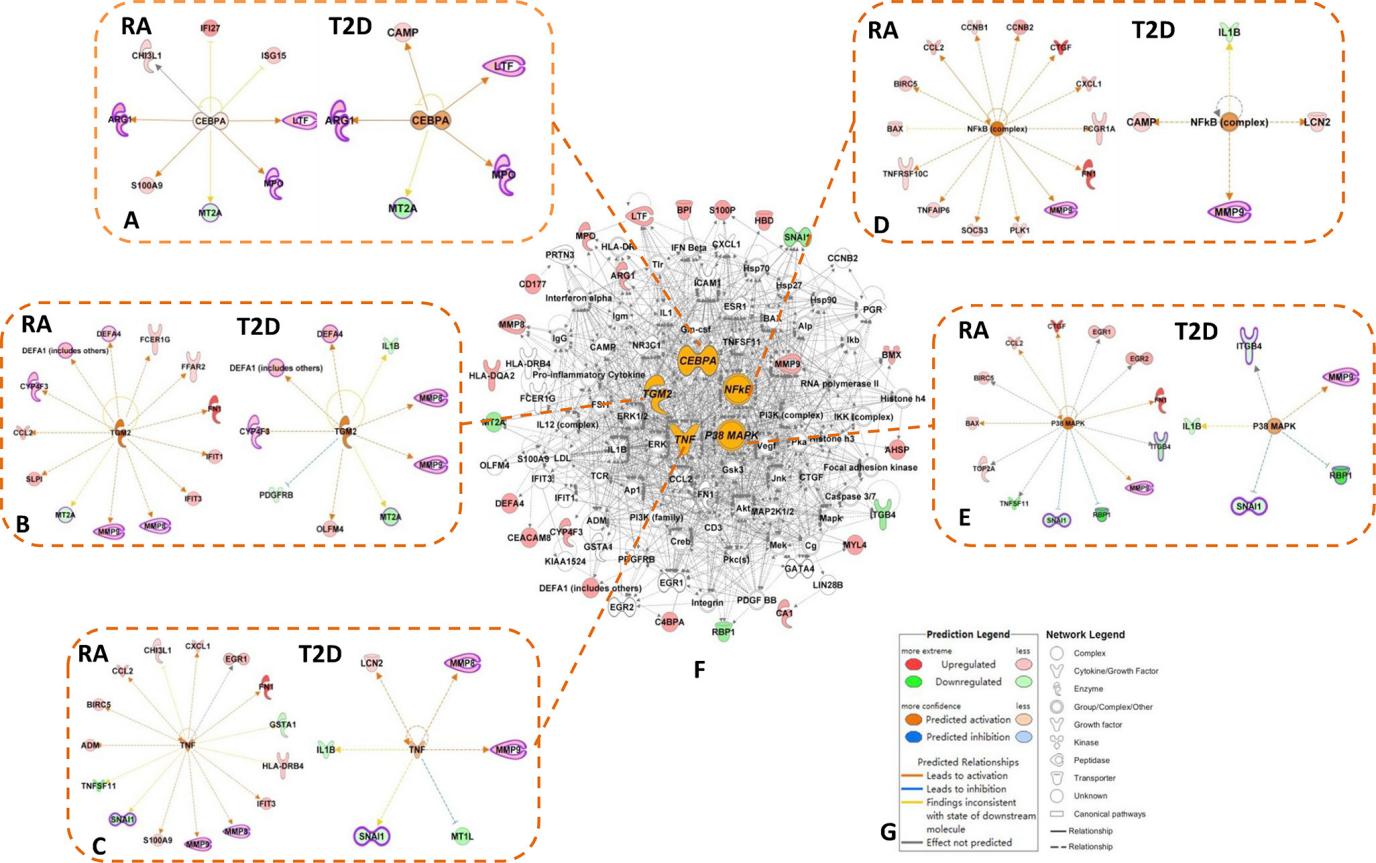
The shared upstream regulators and regulated effects networks between RA and T2D. **A**: The networks of regulator CEBPA and down-stream DEGs in the RA and the T2D groups; **B**: the networks of regulator TGM2 and down-stream DEGs in the RA and the T2D groups; **C**: the networks of regulator TNF and down-stream DEGs in the RA and the T2D groups; **D**: the networks of regulator NF-кB and down-stream DEGs in the RA and the T2D groups; **E**: the networks of regulator p38 MAPK and down-stream DEGs in the RA and the T2D groups; **F**: the shared molecular networks based on the shared DEGs between the RA and the T2D groups. CEBPA, TGM2, TNF, NF-кB and p38 MAPK were the commonly shared upstream regulators both in the RA and the T2D groups, colored with **orange**. **Red nodes**: up-regulated DEGs in RA *vs*. control or T2D *vs*. control groups; **green nodes**: down-regulated DEGs in RA *vs*. control or T2D *vs*. control groups; **violet circle**: the same shared downstream DEGs in the RA and the T2D groups regulated by those three same regulators.

Based on the IPA analysis platform, five shared upstream regulators were predicted between the RA and the T2D groups: TGM2, NF-кB, p38 MAPK, TNF and CEBPA, all of which were predicted up-regulated in both the RA and the T2D groups, as shown in Fig 4 and Table 3. The regulated effects networks between the shared upstream regulators and target molecules were constructed (Fig 4). Among these networks, several shared down-stream DEGs were regulated in both RA and T2D, including CYP4F3, DEFA4, DEFA1, MMP8, MMP9, MT2A, ITGB4, RBP1, SNAI1, ARG1, MPO and LTF. The top bio-functions of the regulated effects networks by 5 regulators were very similar in both RA and T2D (Table 3), concentrated on inflammation, or immune related processes.

**Table 3:** The shared upstream regulators and downstream molecules in RA and T2D and top functions of regulators networks.

Shared upstream regulators	Activation Z-score		Downstream molecules involved in		Top functions of upstream regulator networks	
	RA	T2D	RA	T2D	RA	T2D
***TGM2***	3.06	1.74	***CYP4F3↑***, ***DEFA4↑***, ***DEFA1↑***, ***MMP8↑***, ***MMP9↑***, ***MT2A***↓, CCL2***↑***, SLPI***↑***, FCER1G***↑***, FFAR2***↑***, FN1***↑***, IFIT1***↑***, IFIT3***↑***	***CYP4F3↑***, ***DEFA4↑***, ***DEFA1↑***, ***MMP8↑***, ***MMP9↑***, ***MT2A***↓, IL1β↓, OLFM4***↑***, PDGFRB***↑***	Inflammatory response, immunological disease, skeletal and muscular disorders	Inflammatory disease, skeletal and muscular disorders, cellular movement
***NF-кB***	2.97	1.17	***MMP9↑***, BAX***↑***, BIRC5***↑***, CCL2***↑***, CCNB1***↑***, CCNB2***↑***, CTGF***↑***, CXCL1***↑***, FCGR1A***↑***, FN1***↑***, PLK1***↑***, SOCS3***↑***, TNFAIP6***↑***, TNFRSF10C***↑***	***MMP9↑***, CAMP***↑***, IL1β↓, LCN2***↑***	Cellular development, cellular growth and proliferation, tissue morphology	Cell-to-cell signaling and interaction, cellular movement, immune cell trafficking
***p38MAPK***	2.50	0.98	***ITGB4↓***, ***MMP9↑***, ***RBP1↓***, ***SNAI1↓***, BAX***↑***, BIRC5***↑***, CCL2***↑***, CTGF***↑***, EGR1***↑***, EGR2***↑***, FN1***↑***, TNFSF11↓, TOP2A***↑***	***ITGB4↓***, ***MMP9↑***, ***RBP1↓***, ***SNAI1↓***, IL1β↓	Organismal injury and abnormalities, cellular movement, cell death and survival	Cellular development, Organismal injury and abnormalities, cell death and survival
***TNF***	1.24	0.75	***MMP8↑***, ***MMP9↑***, ***SNAI1***↓, FN1***↑***, CCL2***↑***, IFIT3***↑***, EGR1***↑***, CXCL1***↑***, HLA-DRB4***↑***, ADM***↑***, BIRC5***↑***, S100A9***↑***, CHI3L1***↑***, GSTA1↓, TNFSF11↓	***MMP8***↑, ***MMP9***↑, ***SNAI1***↓, LCN2***↑***, IL1β↓, MT1L***↑***	Cellular movement, inflammatory response, connective tissue disorders	Cellular movement, immune cell trafficking, inflammatory response
***CEBPA***	0.174	1.00	***ARG1↑***, ***MPO↑***, ***MT2A***↓, ***LTF↑***, IFI27***↑***, ISG15***↑***, S100A9***↑***, CHI3L1***↑***	***ARG1↑***, ***MPO↑***, ***MT2A***↓, ***LTF↑***	Cell death and survival, inflammatory response, tissue development	Cell death and survival, cellular movement, immune cell trafficking

### The shared genes verified by Q-PCR

**Fig 5:**
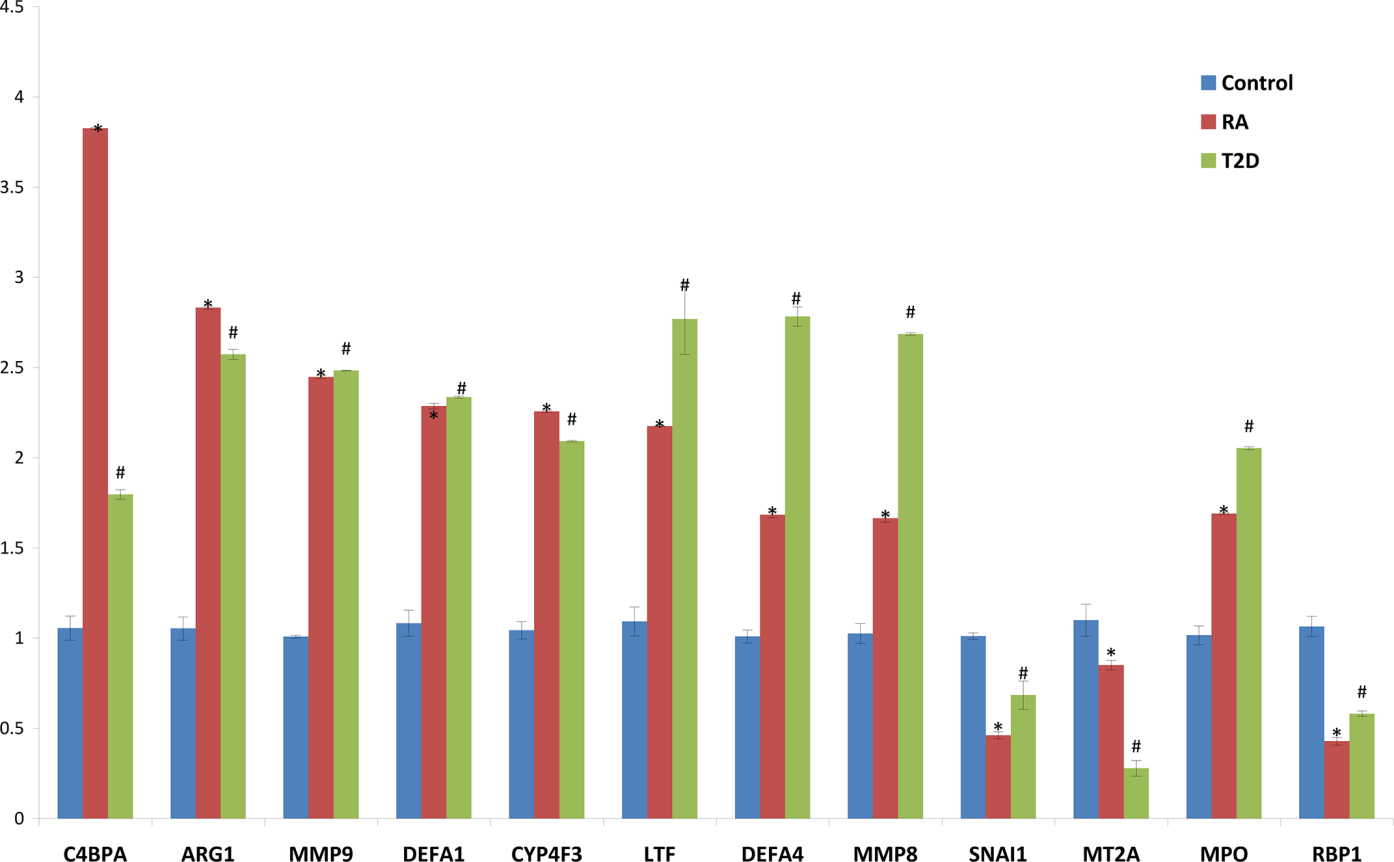
The relative gene expression comparison of the shared top 12 gene of PBMC in healthy control, RA and T2D patients by Quantitative real-time PCR. *: Compared with healthy control, the significant change in RA group, *p*<0.01. #: Compared with healthy control, the significant change in T2D group, *p*<0.01

We chose 12 genes for Q-PCR verification that were differentially expressed in RA and T2D. These genes were C4BPA, ARG1, MMP9, DEFA1, CYP4F3, LTF, MPO, DEFA4, MMP8, RBP1, SNAI1, and MT2A. Six genes were significantly up-regulated in both RA and T2D patients compared with healthy control (*p*-value < 0.01) (Fig 5): C4BPA, ARG1, MMP9, DEFA1, CYP4F3, LTF, MPO, DEFA4 and MMP8. At the same time, the other three genes: RBP1, SNAI1 and MT2A were down-regulated in both RA and T2D patients compared with healthy control (*p*-value < 0.01). The results of Q-PCR were similar tendency with the digital gene expression profile in these groups.

## Discussion

Several studies reported the associations between RA and T2D, but the overall molecular pathways and network associations between the two disease conditions have not been established. This analysis of PBMC gene profiles in RA, T2D and healthy persons by DGE showed the unique and shared gene expression signatures to explain the associations of the two chronic complex diseases further at the transcriptome level.

**Fig 6:**
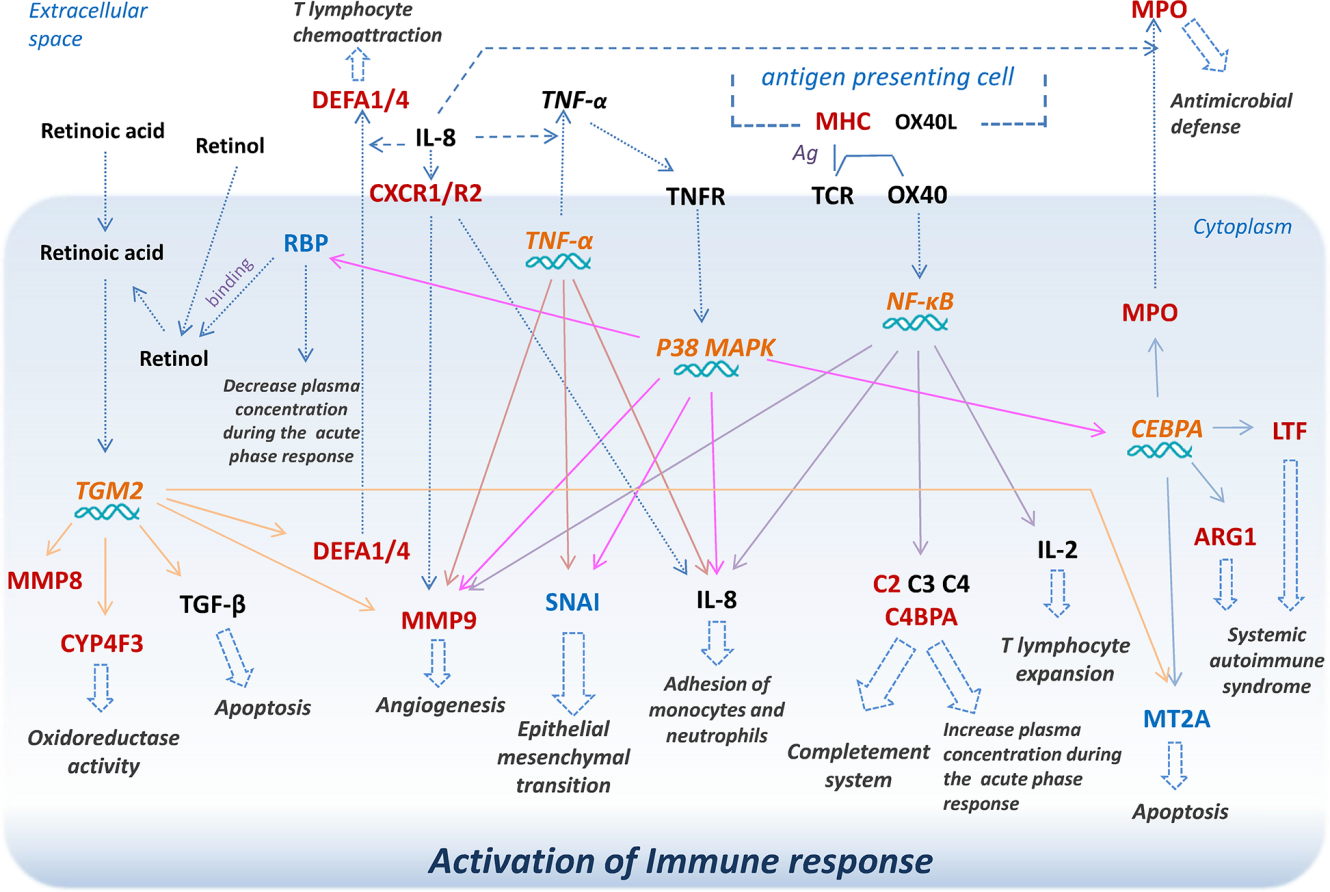
The summary view of shared pathways related with shared DEGs in the RA and the T2D groups involved in immune response. **The red color molecules**: the shared DEGs up-regulated in both RA *vs*. control and T2D *vs*. control groups; **the blue color molecules**: the shared DEGs down-regulated in both RA *vs*. control and T2D *vs*. control groups; **the orange color molecules with italic typ**e: the shared 5 upstream regulators in both RA *vs*. control and T2D *vs*. control groups.

We identified 212 DEGs in RA patients and 114 DEGs in T2D patients compared with healthy volunteers, respectively. 32 DEGs commonly shared in DGE profiles of both RA *vs*. control and T2D *vs*. control groups. Based on the shared top 10 signaling pathways, top 10 bio-functions, 5 predicted upstream regulators, and the shared DEGs involved in, we summarized the shared molecular paths between RA and T2D related with immune response (shown in Fig 6). Five shared upstream regulators TGM2, NF-кB, p38 MAPK, TNF and CEBPA in RA and T2D were predicted as the central regulators in this shared molecular paths. The shared signaling pathways especially complement system, agranulocyte adhesion and diapedesis, IL-8 signaling, Natural killer cell signaling, and differential regulation of cytokine production in intestinal epithelial cells by IL-17A and IL-17F were statistically significant in both RA and T2D. The key shared DEGs in RA and T2D including: CYP4F3, CXCR1/CXCR2, C4BPA, DEFA4, DEFA1, MMP8, MMP9, LTF, ARG1, MT2A, RBP1, MPO, MT2A, SNAI1 and MHC. These genes were regulated by the 5 upstream regulators within interactions and participated in several biological processes, preferentially in the pathways of immune response. The shared molecular pathway and DEGs may be considered as new targets or target networks for the prevention and treatment of patients with RA and T2D simultaneously.

The complement system constitutes an important component of the defense against foreign organisms, functioning both in innate and adaptive immune systems, which is potentially harmful also to the own organism [35]. There was strong evidence implied that both the classical and the alternative pathways of complement are pathologically activated during RA [36]. There was also a high level of complements in both types of diabetes mellitus [37, 38]. Same with other reports, the complement system was found as one of the most important shared signaling pathway between RA and T2D in this study. The up-regulated DEG C4BPA participated in complement system signaling, indicated the activation of complement system in both RA and T2D. C4b-binding protein (C4BP) is the most important soluble regulator of the classical pathway of complement activation, one major form of which is C4BP-alph (C4BPA) [39]. In vitro studies demonstrate that IL-6, IL-1 beta, and IFN-gamma increase the levels of C4BPA mRNAs [39]. In our finding, C4BPA were up-regulated both in RA and T2D, may indicated the interaction of lots of inflammatory cytokines during both the two disorders. C4BPA was also involved in acute phase response signaling in both RA and T2D in this study, which reported that the highly elevated levels of acute phase proteins at the onset of RA [40]. We found acute phase response signaling also related with T2D patients, with shared DEGs C4BPA and RBP1, which maybe a novel finding of T2D pathological mechanism.

Agranulocyte (lymphocytes and monocytes) adhesion and diapedesis were also found shared in RA and T2D, which is a key event in the process of inflammation and cellular immune response. Agranulocytes response to inflammatory signals such as TNFs, interleukins, complement components and histamine, and then diapedesis passing between neighboring endothelial cells (transmigration) to reach the infected tissues [41]. Activated MMPs, such as MMP8 and MMP9, which were up-regulated in both RA and T2D in our study, can degrade the assembly of junctional proteins, leading to the opening of inter-endothelial cell contacts, allowing agranulocytes to transmigrate between adjacent endothelial cells to reach the underlying tissue [42]. Many MMPs are expressed at increased levels in RA tissues and in synoviocyte cultures in response to inflammatory cytokines including MMP8, MMP9 [43]. There were evidences for the association of MMP9 with type 2 diabetes, but not the case for MMP8 [44]. Up-regulated MMP8 expression in PBMC in T2D patients may provide novel point in understanding T2D.

The IL-8 signaling pathway was also identified commonly shared between the RA and the T2D in this study, in which 3 identified DEGs were in common: DEFA1, MMP9, and MPO. Significant evidence implicates IL-8 as a major mediator of inflammation and joint destruction in rheumatoid arthritis [45]. In response to inflammatory events, activated neutrophils release myeloperoxidase (MPO), which is an enzyme producing hypohalous acids for the microbicidal activity of neutrophils [46]. Consistent with previous research, MPO were up-regulated in both RA and T2D patients in our study [46, 47]. In addition, we found the induction of the Defensin gene: DEFA1/HNP-1 (defensin, alpha 1) which was also up-regulated in both RA and T2D in the current study. DEGs DEFA1/4 was a member of the family of microbicidal and cytotoxic peptides involved in host defense associated with RA [48]. It is reported that up-regulation of human DEFA1 mRNA in bone marrow-derived mononuclear cells is associated with rheumatoid arthritis in human [49]. And others found that DEFA had a significantly higher expression in RA patients than in healthy controls from total RNA of PBMC [48]. However, we did not find reports about the role that DEFA plays in the pathological process of T2D. In our study, DEFA1/4 was up-regulated both in RA and T2D, involved in IL-8 signaling. This phenomenon revealed that DEFA1 might be a novel gene in T2D patients, and could be a potential biomarker in both RA and T2D. Except of agranulocyte adhesion and diapedesis pathways, MMP9 was also up-regulated in both the RA and the T2D groups involved in IL-8 signaling, which can lead to an inflammatory response through the regulation of endothelial cell migration during angiogenesis [50, 51]. In this point, we speculated that the up-regulated MMP9 involved in cytokine and cellular immune response signaling may be the aetiopathogenesis commonality of RA and T2D. Furthermore, we know IL-1β perpetuate Th17 responses and endothelial cell damage, which potentiate the rheumatoid arthritis[52]. IL-1β has important roles in endocrinology and in the regulation of responses associated with inflammatory stress. IL-1 impairs insulin secretion and induces β-cell apoptosis leading to T2D[53]. IL-8 is strongly dependent of IL-1beta and the level of IL-1 beta in RA and T2D remained unchanged in our study. it is elevated in other tissues and maybe the cause of the increase in IL-8 in the circulating immune cells. For the two shared pathways: natural killer cell signaling and differential regulation of cytokine production in intestinal epithelial cells by IL-17A and IL-17F, they were also found in both RA and T2D. Natural killer cells are large granular lymphocytes that are important to the innate immune system, which have the capacity to damage normal cells or through interaction with other cells such as dendritic cells, macrophages, and T cells cause autoimmune diseases, such as RA [54]. And natural killer cells were reported associated with obesity and other metabolic diseases, such as diabetes [55]. However, in our study, we didn’t find the shared DEGs involved in natural killer cells signaling between these two disorders, although it was the commonly shared pathway in both RA and T2D. IL-17A and IL-17F are primarily produced by a subset of T cells called Th17 and are highly homologous, which have been linked to cytokine and chemokine production in various inflammatory and/or autoimmune diseases, such as RA [56]. IL-17A and IL-17F was reported elevated in the synovial tissues or plasma of patients with RA [57, 58] and inhibited adipocyte differentiation in T2D [18]. Differential regulation of cytokine production in intestinal epithelial cells by IL-17A and IL-17F was found shared in both RA and T2D in our study, even without shared DEGs. In this point, we speculated this two pathways involved in immune response was the aetiopathogenesis commonality of RA and T2D, which needs further study for verification.

Furthermore, 5 upstream regulators predicted to be activated in both RA and T2D in this study, and the common networks shared by them between RA and T2D were identified by the IPA platform: TGM2, NF-кB, p38 MAPK, TNF and CEBPA (Fig 4, Table 3). Most of the shared DEGs were regulated by these 5 regulators leading to the immune response at the end, such as CYP4F3, DEFA4, DEFA1, MMP8, MMP9, MT2A, ITGB4, RBP1, SNAI1, ARG1, MPO and LTF. Among these genes, MT2A, ITGB4, RBP1 and SNAI1 were down-regulated, the others were up-regulated. TGM2, transglutaminase 2, was the most important regulators in this study with the highest activation z-score among all the upstream regulators in both RA and T2D (z-score means–log10 of the Fisher’ Exact test *p*-value statistics by IPA). As a multi-functional enzyme, the protein encoded by this gene acts as a monomer, which is induced by retinoic acid and appears to be involved in apoptosis [59]. Missense mutations in the TGM2 gene encoding transglutaminase 2 are found in patients with early-onset type 2 diabetes [59], but little reports found the relation between TGM2 and RA. As we know, regulators p38 MAPK, TNF and NF-кB play important roles in transducing inflammation, by which several transcription factors can be directly phosphorylated and activated to bring about pro-inflammatory factors in RA, T2D and other inflammatory or immune diseases, and some of them had already been used as drug targets curing RA or T2D, such as the TNF inhibitors [60–64]. The p38MAPK has been considered a promising target for development of new anti-inflammatory drugs to treat RA [65]. It is also reported that inhibition of the human p38MAPK protein(s) decreases palmitate-induced insulin resistance of HepG2 cells in cell culture [66], suggesting that regulation of p38MAPK may play a role in treatment of T2D. CEBPA, CCAAT/enhancer binding protein alpha (C/EBPα), was predicted shared in both RA and T2D as another upstream regulator, to control the activity of ARG1, MPO, MT2A and LTF downstream. CEBPA is a transcription factor that influences immune cell fate and differentiation, the activity of this protein can modulate the expression of genes involved in cell cycle regulation and in body weight homeostasis[67]. Some researchers found the high expression level of CEBPA in RA and other inflammatory disorders, which may with other important transcription factors, such as p38MAPK, NF-кB and TNF [68, 69]. In addition, homozygous mutant mouse Cebpa gene (knockout) increases hypoinsulinemia in mouse participated in metabolic processes [70]. Because the bio-functions of CEBPA related with RA or T2D was not yet fully understood, there were few agents reported for curing of RA or T2D. Thus, CEBPA may be considered as a novel target for treatment of these two disorders.

In summary, from the shared path (Fig 6) in RA and T2D, we found the shared key DEGs were regulated by 5 shared upstream regulators, involved in shared key signaling pathways and important functional processes, leading to multiple immune response paths. Based on these genes and pathways, we constructed the molecular networks to show the potential interactions and cross-talking between these pathways, which was considered as novel insights for the understanding of the disorders, and may be possible to help to discover new targets for the treatment of these two diseases.

It is need to point out that obesity is associated with a low-grade inflammation, which influences adipocyte function and may promote type 2 diabetes[71]. the patients with type 2 diabetes included in this study are much leaner compared to European and even more USA patients with type 2 diabetes. Our results more reflect the basic situation of the Han people.

## Limitations

There were still limitations of this study. The number of patients incorporated into the study was not large enough to come to definite conclusions. The results of DGE need to be verified by further experiments and clinical indexes. In addition, the patients with situation of coexist of both RA and T2D disorders were not included in the study, because of the limitation of research conditions. Therefore, we may provide novel view of commonly shared molecular mechanism and predicting the potential markers of RA and T2D, but still need further research deeply and exactly.

## Conclusions

In order to find the associations between RA and T2D, we preformed RNA sequencing approach on DGE results. We identified 212 DEGs in RA patients and 114 DEGs in T2D patients compared with healthy volunteers. 32 shared DEGs commonly shared in DGE profiles of RA and T2D comparison. Based on the shared top 10 signaling pathways, top 10 bio-functions, 5 predicted upstream regulators, and the shared DEGs involved in, we summarized the shared molecular paths between RA and T2D related to activation of immune response. Five shared upstream regulators TGM2, NF-кB, p38 MAPK, TNF and CEBPA in RA and T2D were predicted as the central regulators in the shared molecular paths. The shared signaling pathways especially complement system, agranulocyte adhesion and diapedesis, IL-8 signaling, natural killer cell signaling, and differential regulation of cytokine production in intestinal epithelial cells by IL-17A and IL-17F were statistically significant in both RA and T2D. The key shared DEGs in RA and T2D were regulated by the upstream regulators and interactions, participated in several biological processes most of which are involved in immune response. Those discoveries maybe considered as new understanding of the associations of RA and T2D and provided some new insights for treatment targets of the two diseases, especially as novel early prevention methods.

## Supporting Information

S1 File Contains Table A. The identified differently expressed genes in RA *vs*. Control, Table B. The identified differently expressed genes in T2D *vs*. Control, Table C. The commonly shared identified differently expressed genes between RA *vs*. Control and T2D *vs*. Control.
(DOC)
