# Supplementary material for: The Crosstalk of Pathways Involved in Immune Response Maybe the Shared Molecular Basis of Rheumatoid Arthritis and Type 2 Diabetes
Source: PLoS One. 2015 Aug 7;10(8):e0134990. doi: 10.1371/journal.pone.0134990 (PMC4529222; doi:10.1371/journal.pone.0134990)
Supplement: S1 File — The identified differently expressed genes in RA vs. Control, Table B. The identified differently expressed genes in T2D vs. Control, Table C. The commonly shared identified differently expressed genes between RA vs. Control and T2D vs. Control. (DOC) [file pone.0134990.s001.doc]

**Supporting Tables:**

**Supporting Table A. The identified differently expressed genes in RA *vs.* Control**

| **No.** | **Gene Symbol** | **ID** | **Ratio** | ***P*-value** |
| --- | --- | --- | --- | --- |
| ***1*** | ***MIR508*** | NR_030235 | 5.64 | 1.36E-09 |
| ***2*** | ***T*** | NM_003181 | 5.17 | 3.78E-07 |
| ***3*** | ***KIR2DS5*** | NM_014513 | 5.09 | 7.65E-07 |
| ***4*** | ***BEX2*** | NM_001168399 | 5.04 | 1.55E-06 |
| ***5*** | ***GBP1P1*** | NR_003133 | 4.95 | 3.12E-06 |
| ***6*** | ***CTGF*** | NM_001901 | 4.95 | 3.12E-06 |
| ***7*** | ***ASPRV1*** | NM_152792 | 4.86 | 6.31E-06 |
| ***8*** | ***SNCB*** | NM_001001502 | 4.81 | 1.28E-05 |
| ***9*** | ***RNF17*** | NM_031277 | 4.81 | 1.28E-05 |
| ***10*** | ***FN1*** | NM_212478 | 4.71 | 1.72E-05 |
| ***11*** | ***ZKSCAN4*** | NM_019110 | 4.70 | 2.58E-05 |
| ***12*** | ***METTL7B*** | NM_152637 | 4.58 | 5.21E-05 |
| ***13*** | ***MET*** | NM_000245 | 4.46 | 1.05E-04 |
| ***14*** | ***LOC100132215*** | NR_033389 | 4.39 | 2.13E-04 |
| ***15*** | ***TBX15*** | NM_152380 | 4.39 | 2.13E-04 |
| ***16*** | ***LOC647012*** | NR_033658 | 4.39 | 2.13E-04 |
| ***17*** | ***BMX*** | NM_001721 | 4.05 | 7.58E-05 |
| ***18*** | ***FCGR3B*** | NM_000570 | 3.92 | 1.79E-06 |
| ***19*** | ***RNF182*** | NM_001165034 | 3.46 | 7.60E-05 |
| ***20*** | ***C4BPA*** | NM_000715 | 3.32 | 1.38E-11 |
| ***21*** | ***SLC26A8*** | NM_001193476 | 3.02 | 1.79E-06 |
| ***22*** | ***BTNL3*** | NM_197975 | 3.00 | 9.97E-06 |
| ***23*** | ***C1QC*** | NM_172369 | 3.00 | 1.35E-13 |
| ***24*** | ***HBZ*** | NM_005332 | 2.89 | 5.05E-05 |
| ***25*** | ***PRDM16*** | NM_022114 | 2.89 | 5.05E-05 |
| ***26*** | ***IFI27*** | NM_001130080 | 2.76 | 0.00 |
| ***27*** | ***AEBP1*** | NM_001129 | 2.64 | 4.62E-05 |
| ***28*** | ***STOX1*** | NM_001130159 | 2.58 | 1.93E-05 |
| ***29*** | ***KCNMA1*** | NM_001014797 | 2.57 | 5.45E-07 |
| ***30*** | ***OTOF*** | NM_194322 | 2.53 | 9.50E-14 |
| ***31*** | ***EGR2*** | NM_001136178 | 2.50 | 0.00 |
| ***32*** | ***LOC642236*** | NR_033907 | 2.44 | 1.13E-05 |
| ***33*** | ***CD177*** | NM_020406 | 2.40 | 0.00 |
| ***34*** | ***CCNB2*** | NM_004701 | 2.39 | 1.26E-13 |
| ***35*** | ***C7orf25*** | NM_024054 | 2.37 | 8.04E-06 |
| ***36*** | ***C1QB*** | NM_000491 | 2.36 | 0.00 |
| ***37*** | ***NOV*** | NM_002514 | 2.35 | 2.98E-07 |
| ***38*** | ***NOP16*** | NM_016391 | 2.33 | 4.74E-08 |
| ***39*** | ***SIGLEC1*** | NM_023068 | 2.32 | 5.68E-13 |
| ***40*** | ***CNTNAP3*** | NM_033655 | 2.27 | 2.12E-05 |
| ***41*** | ***CA4*** | NM_000717 | 2.26 | 1.68E-10 |
| ***42*** | ***ARG1*** | NM_000045 | 2.22 | 3.89E-14 |
| ***43*** | ***PSMD4*** | NM_002810 | 2.20 | 1.71E-4 |
| ***44*** | ***SLPI*** | NM_003064 | 2.18 | 2.64E-06 |
| ***45*** | ***SPESP1*** | NM_145658 | 2.16 | 3.20E-06 |
| ***46*** | ***IGFL2*** | NM_001002915 | 2.16 | 3.71E-05 |
| ***47*** | ***MME*** | NM_007287 | 2.11 | 0.00 |
| ***48*** | ***ADA*** | NM_000022 | 2.08 | 2.31E-12 |
| ***49*** | ***CCL2*** | NM_002982 | 2.07 | 0.00 |
| ***50*** | ***BPI*** | NM_001725 | 2.06 | 8.22E-06 |
| ***51*** | ***HSPA6*** | NM_002155 | 2.06 | 0.00 |
| ***52*** | ***CNTNAP3B*** | NM_001201380 | 2.02 | 3.27E-06 |
| ***53*** | ***PLIN4*** | NM_001080400 | 2.02 | 3.32E-10 |
| ***54*** | ***CCR9*** | NM_006641 | 1.99 | 9.50E-14 |
| ***55*** | ***DLGAP5*** | NM_014750 | 1.96 | 3.29E-08 |
| ***56*** | ***CXCR1*** | NM_000634 | 1.96 | 0.00 |
| ***57*** | ***HBG1*** | NM_000559 | 1.95 | 0.00 |
| ***58*** | ***IFIT3*** | NM_001031683 | 1.92 | 0.00 |
| ***59*** | ***UHRF1*** | NM_001048201 | 1.92 | 8.97E-10 |
| ***60*** | ***PGLYRP1*** | NM_005091 | 1.91 | 0.00 |
| ***61*** | ***MMP9*** | NM_004994 | 1.90 | 2.72E-13 |
| ***62*** | ***TPX2*** | NM_012112 | 1.90 | 0.00 |
| ***63*** | ***FOLR3*** | NM_000804 | 1.85 | 0.00 |
| ***64*** | ***ALPL*** | NM_000478 | 1.85 | 3.55E-15 |
| ***65*** | ***DEFA3*** | NM_005217 | 1.85 | 1.92E-12 |
| ***66*** | ***KCNJ2*** | NM_000891 | 1.85 | 2.24E-13 |
| ***67*** | ***EGR1*** | NM_001964 | 1.84 | 1.36E-11 |
| ***68*** | ***RPL37A*** | NM_000998 | 1.83 | 0.00 |
| ***69*** | ***CXCL1*** | NM_001511 | 1.82 | 0.00 |
| ***70*** | ***IFIT1*** | NM_001548 | 1.81 | 4.87E-12 |
| ***71*** | ***ANKRD22*** | NM_144590 | 1.78 | 0.00 |
| ***72*** | ***CYP4F3*** | NM_001199209 | 1.77 | 0.00 |
| ***73*** | ***RNASE1*** | NM_198232 | 1.75 | 3.79E-09 |
| ***74*** | ***HLA-DRB4*** | NM_021983 | 1.71 | 0.00 |
| ***75*** | ***KRT23*** | NM_015515 | 1.71 | 0.00 |
| ***76*** | ***LTF*** | NM_001199149 | 1.71 | 0.00 |
| ***77*** | ***ASPM*** | NM_018136 | 1.71 | 0.00 |
| ***78*** | ***C17orf56*** | NM_144679 | 1.70 | 0.00 |
| ***79*** | ***C5orf4*** | NM_032385 | 1.67 | 0.00 |
| ***80*** | ***KCNJ15*** | NM_002243 | 1.66 | 5.49E-13 |
| ***81*** | ***AHSP*** | NM_016633 | 1.65 | 0.00 |
| ***82*** | ***FHDC1*** | NM_033393 | 1.63 | 5.52E-09 |
| ***83*** | ***SIGLEC5*** | NM_003830 | 1.63 | 6.99E-08 |
| ***84*** | ***MCM10*** | NM_018518 | 1.63 | 2.06E-06 |
| ***85*** | ***CDT1*** | NM_030928 | 1.62 | 0.00 |
| ***86*** | ***C8orf38*** | NM_152416 | 1.62 | 1.17E-13 |
| ***87*** | ***ADM*** | NM_001124 | 1.61 | 0.00 |
| ***88*** | ***C19orf71*** | NM_001135580 | 1.60 | 9.72 E-2 |
| ***89*** | ***BIRC5*** | NM_001168 | 1.60 | 4.75E-14 |
| ***90*** | ***BATF2*** | NM_138456 | 1.59 | 5.15E-14 |
| ***91*** | ***MYL4*** | NM_002476 | 1.59 | 0.00 |
| ***92*** | ***RPGRIP1*** | NM_020366 | 1.58 | 4.82E-06 |
| ***93*** | ***BAMBI*** | NM_012342 | 1.58 | 4.82E-06 |
| ***94*** | ***MND1*** | NM_032117 | 1.58 | 1.02E-06 |
| ***95*** | ***PBK*** | NM_018492 | 1.58 | 5.29E-08 |
| ***96*** | ***TIMD4*** | NM_001146726 | 1.56 | 7.33E-06 |
| ***97*** | ***CEP55*** | NM_001127182 | 1.55 | 2.14E-07 |
| ***98*** | ***FAM154B*** | NM_001008226 | 1.55 | 0.00 |
| ***99*** | ***IFI44L*** | NM_006820 | 1.54 | 0.00 |
| ***100*** | ***HARBI1*** | NM_173811 | 1.54 | 2.29 E-5 |
| ***101*** | ***MACROD2*** | NM_080676 | 1.54 | 5.15E-14 |
| ***102*** | ***IFITM3*** | NM_021034 | 1.53 | 0.00 |
| ***103*** | ***ISG15*** | NM_005101 | 1.52 | 0.00 |
| ***104*** | ***KIAA0101*** | NM_001029989 | 1.52 | 0.00 |
| ***105*** | ***MMP25*** | NM_022468 | 1.52 | 5.90E-12 |
| ***106*** | ***BTNL8*** | NM_001159708 | 1.51 | 4.61E-06 |
| ***107*** | ***KREMEN1*** | NM_001039570 | 1.50 | 1.02E-11 |
| ***108*** | ***FCGR1A*** | NM_000566 | 1.50 | 0.00 |
| ***109*** | ***SKA1*** | NM_001039535 | 1.50 | 1.96E-05 |
| ***110*** | ***PHOSPHO1*** | NM_001143804 | 1.48 | 7.39E-14 |
| ***111*** | ***USP18*** | NM_017414 | 1.48 | 2.26E-13 |
| ***112*** | ***FCER1G*** | NM_004106 | 1.48 | 8.50E-13 |
| ***113*** | ***CMTM2*** | NM_001199317 | 1.47 | 0.00 |
| ***114*** | ***S100P*** | NM_005980 | 1.47 | 3.13E-12 |
| ***115*** | ***RSAD2*** | NM_080657 | 1.47 | 2.25E-13 |
| ***116*** | ***TOP2A*** | NM_001067 | 1.46 | 0.00 |
| ***117*** | ***PRKCDBP*** | NM_145040 | 1.45 | 5.32E-07 |
| ***118*** | ***TYMS*** | NM_001071 | 1.43 | 5.81E-13 |
| ***119*** | ***IL1R2*** | NM_173343 | 1.43 | 0.00 |
| ***120*** | ***CDC6*** | NM_001254 | 1.42 | 2.57E-08 |
| ***121*** | ***KIF4A*** | NM_012310 | 1.42 | 3.28E-07 |
| ***122*** | ***LRG1*** | NM_052972 | 1.42 | 6.96E-13 |
| ***123*** | ***CXCR2*** | NM_001168298 | 1.41 | 6.81E-12 |
| ***124*** | ***WNT16*** | NM_057168 | 1.40 | 2.73E-08 |
| ***125*** | ***UNC13B*** | NM_006377 | 1.40 | 3.04E-06 |
| ***126*** | ***HBD*** | NM_000519 | 1.40 | 1.79E-13 |
| ***127*** | ***BAX*** | NR_027882 | 1.39 | 2.01E-13 |
| ***128*** | ***ANXA3*** | NM_005139 | 1.37 | 1.41E-13 |
| ***129*** | ***SIGLEC14*** | NM_001098612 | 1.36 | 0.00 |
| ***130*** | ***SOCS3*** | NM_003955 | 1.36 | 2.72E-12 |
| ***131*** | ***CES1*** | NM_001025194 | 1.35 | 0.00 |
| ***132*** | ***RPH3A*** | NM_001143854 | 1.35 | 1.03E-13 |
| ***133*** | ***CDKN3*** | NM_001130851 | 1.34 | 0.00 |
| ***134*** | ***CCNB1*** | NM_031966 | 1.34 | 8.34E-10 |
| ***135*** | ***S100A9*** | NM_002965 | 1.34 | 1.45E-10 |
| ***136*** | ***C1QA*** | NM_015991 | 1.33 | 2.11E-13 |
| ***137*** | ***OIP5*** | NM_007280 | 1.33 | 9.17E-07 |
| ***138*** | ***DIRAS1*** | NM_145173 | 1.32 | 6.47E-07 |
| ***139*** | ***MOSC1*** | NM_022746 | 1.31 | 1.00E-13 |
| ***140*** | ***HLA-DQA2*** | NM_020056 | 1.30 | 3.75E-12 |
| ***141*** | ***PLK1*** | NM_005030 | 1.30 | 0.00 |
| ***142*** | ***KIR2DL4*** | NM_001080772 | 1.30 | 3.02E-14 |
| ***143*** | ***IFI44*** | NM_006417 | 1.30 | 1.87E-11 |
| ***144*** | ***LIN7A*** | NM_004664 | 1.30 | 0.00 |
| ***145*** | ***C2*** | NM_001145903 | 1.30 | 3.80E-13 |
| ***146*** | ***CMPK2*** | NM_207315 | 1.29 | 1.14E-12 |
| ***147*** | ***SERINC2*** | NM_001199038 | 1.29 | 1.26E-11 |
| ***148*** | ***BUB1B*** | NM_001211 | 1.29 | 2.48E-12 |
| ***149*** | ***PI4KA*** | NM_058004 | 1.28 | 0.00 |
| ***150*** | ***LETM2*** | NM_144652 | 1.28 | 5.10E-05 |
| ***151*** | ***C5orf32*** | NM_032412 | 1.28 | 2.94E-13 |
| ***152*** | ***CPNE9*** | NM_153635 | 1.28 | 2.05E-05 |
| ***153*** | ***KIF11*** | NM_004523 | 1.27 | 0.00 |
| ***154*** | ***MGAM*** | NM_004668 | 1.27 | 3.53E-13 |
| ***155*** | ***PPIL6*** | NM_173672 | 1.27 | 0.00 |
| ***156*** | ***SELENBP1*** | NM_003944 | 1.26 | 0.00 |
| ***157*** | ***GINS2*** | NM_016095 | 1.26 | 6.63E-07 |
| ***158*** | ***CHI3L1*** | NM_001276 | 1.26 | 4.44E-11 |
| ***159*** | ***ZNF202*** | NM_003455 | 1.26 | 4.44E-10 |
| ***160*** | ***CDC45*** | NM_003504 | 1.26 | 6.37E-09 |
| ***161*** | ***TNFAIP6*** | NM_007115 | 1.25 | 2.12E-13 |
| ***162*** | ***TNFRSF10C*** | NM_003841 | 1.25 | 1.85E-13 |
| ***163*** | ***HMMR*** | NM_001142557 | 1.25 | 3.10E-11 |
| ***164*** | ***INSL3*** | NM_005543 | 1.24 | 0.00 |
| ***165*** | ***CA1*** | NM_001128830 | 1.24 | 1.23E-13 |
| ***166*** | ***UBB*** | NM_018955 | 1.24 | 0.00 |
| ***167*** | ***MX1*** | NM_002462 | 1.24 | 0.00 |
| ***168*** | ***ACAA1*** | NR_024024 | 1.24 | 0.00 |
| ***169*** | ***MPO*** | NM_000250 | 1.23 | 0.00 |
| ***170*** | ***FFAR2*** | NM_005306 | 1.23 | 2.53E-12 |
| ***171*** | ***ESPL1*** | NM_012291 | 1.23 | 8.24E-06 |
| ***172*** | ***LRRC4*** | NM_022143 | 1.23 | 0.00 |
| ***173*** | ***PRUNE2*** | NM_015225 | 1.23 | 2.04E-08 |
| ***174*** | ***NCRNA00256A*** | NR_024366 | 1.22 | 4.49E-08 |
| ***175*** | ***DEFA4*** | NM_001925 | 1.22 | 2.15E-10 |
| ***176*** | ***DHRS9*** | NM_005771 | 1.21 | 0.00 |
| ***177*** | ***FAM177B*** | NM_207468 | 1.21 | 4.76E-06 |
| ***178*** | ***CCNA2*** | NM_001237 | 1.21 | 0.00 |
| ***179*** | ***ANO5*** | NM_213599 | 1.21 | 1.29E-07 |
| ***180*** | ***ABTB2*** | NM_145804 | 1.21 | 8.13E-06 |
| ***181*** | ***MKI67*** | NM_001145966 | 1.21 | 0.00 |
| ***182*** | ***SDC3*** | NM_014654 | 1.20 | 1.48E-10 |
| ***183*** | ***MMP8*** | NM_002424 | 1.20 | 4.47E-07 |
| ***184*** | ***ETV7*** | NM_001207039 | 1.20 | 3.39E-12 |
| ***185*** | ***RBP1*** | NM_002899 | -7.06 | 3.80E-24 |
| ***186*** | ***EFCAB4B*** | NM_032680 | -4.75 | 1.82E-05 |
| ***187*** | ***IGLL3P*** | NR_029395 | -4.35 | 1.02E-20 |
| ***188*** | ***ALS2CR11*** | NM_001168217 | -3.42 | 1.46E-04 |
| ***189*** | ***TNFSF11*** | NM_003701 | -3.00 | 8.27E-07 |
| ***190*** | ***DSCAML1*** | NM_020693 | -2.68 | 2.48E-05 |
| ***191*** | ***ITGB4*** | NM_000213 | -2.48 | 1.21E-07 |
| ***192*** | ***NRARP*** | NM_001004354 | -2.02 | 5.46E-05 |
| ***193*** | ***CDRT15L2*** | NM_001190790 | -1.70 | 2.00E-06 |
| ***194*** | ***SNAI1*** | NM_005985 | -1.67 | 2.05E-11 |
| ***195*** | ***GSTA1*** | NM_145740 | -1.51 | 5.64E-05 |
| ***196*** | ***DAAM1*** | NM_014992 | -1.49 | 1.55E-08 |
| ***197*** | ***SNX7*** | NM_152238 | -1.49 | 3.32E-05 |
| ***198*** | ***CACNG6*** | NM_031897 | -1.44 | 3.79E-05 |
| ***199*** | ***KRT1*** | NM_006121 | -1.42 | 3.11E-06 |
| ***200*** | ***MTRNR2L1*** | NM_001190452 | -1.34 | 7.031E-11 |
| ***201*** | ***CYP1A2*** | NM_000761 | -1.33 | 2.33E-05 |
| ***202*** | ***THEM5*** | NM_182578 | -1.31 | 2.51E-04 |
| ***203*** | ***YEATS2*** | NM_018023 | -1.30 | 8.56E-07 |
| ***204*** | ***LOC100507266*** | NR_037888 | -1.27 | 1.55E-07 |
| ***205*** | ***DDX19A*** | NM_018332 | -1.26 | 8.05E-05 |
| ***206*** | ***TNNC2*** | NM_003279 | -1.26 | 4.94E-07 |
| ***207*** | ***MT2A*** | NM_005953 | -1.25 | 0.00 |
| ***208*** | ***HSD17B3*** | NM_000197 | -1.24 | 2.61E-6 |
| ***209*** | ***TECPR1*** | NM_015395 | -1.24 | 9.11E-15 |
| ***210*** | ***AREG*** | NM_001657 | -1.24 | 6.56E-10 |
| ***211*** | ***CXXC1P1*** | NR_033924 | -1.22 | 1.32E-14 |
| ***212*** | ***HGD*** | NM_000187 | -1.22 | 2.80E-29 |

**Supporting Table B.** **The identified differently expressed genes in T2D *vs.* Control**

| **No.** | **Gene Symbol** | **ID** | **Ratio** | ***P*-value** |
| --- | --- | --- | --- | --- |
| ***1*** | ***RNF17*** | NM_031277 | 5.70 | 4.95E-10 |
| ***2*** | ***SLC44A5*** | NM_001130058 | 5.61 | 1.97E-09 |
| ***3*** | ***RNF182*** | NM_001165034 | 5.29 | 7.31E-05 |
| ***4*** | ***SLC16A10*** | NM_018593 | 4.52 | 6.28E-05 |
| ***5*** | ***EVPL*** | NM_001988 | 3.81 | 1.18E-04 |
| ***6*** | ***BMX*** | NM_001721 | 3.07 | 8.91E-05 |
| ***7*** | ***HIST1H1E*** | NM_005321 | 2.81 | 2.35E-04 |
| ***8*** | ***BTNL3*** | NM_197975 | 2.52 | 9.54E-06 |
| ***9*** | ***GYPA*** | NM_002099 | 2.51 | 1.54E-04 |
| ***10*** | ***CYB5R2*** | NM_016229 | 2.39 | 2.28E-04 |
| ***11*** | ***BPI*** | NM_001725 | 2.39 | 1.61E-06 |
| ***12*** | ***RNU4-2*** | NR_003137 | 2.34 | 8.33E-14 |
| ***13*** | ***PLCH1*** | NM_001130960 | 2.18 | 1.40E-07 |
| ***14*** | ***LOC642236*** | NR_033907 | 2.17 | 5.36E-05 |
| ***15*** | ***LTF*** | NM_001199149 | 2.17 | 0.00 |
| ***16*** | ***SYT6*** | NM_205848 | 2.13 | 4.93E-05 |
| ***17*** | ***SLC26A8*** | NM_001193476 | 2.04 | 7.50E-06 |
| ***18*** | ***DEFA4*** | NM_001925 | 2.03 | 0.00 |
| ***19*** | ***OLFM4*** | NM_006418 | 2.02 | 0.00 |
| ***20*** | ***HBG2*** | NM_000184 | 2.02 | 2.18E-12 |
| ***21*** | ***ARG1*** | NM_000045 | 2.01 | 6.48E-14 |
| ***22*** | ***ADCY6*** | NM_020983 | 2.00 | 4.41E-06 |
| ***23*** | ***AMH*** | NM_000479 | 1.98 | 1.04E-06 |
| ***24*** | ***MMP8*** | NM_002424 | 1.96 | 4.71E-14 |
| ***25*** | ***CEACAM8*** | NM_001816 | 1.96 | 2.56E-13 |
| ***26*** | ***ABCA13*** | NM_152701 | 1.94 | 5.18E-07 |
| ***27*** | ***PRRT4*** | NM_001174164 | 1.93 | 2.28E-06 |
| ***28*** | ***AHSP*** | NM_016633 | 1.93 | 0.00 |
| ***29*** | ***MMP9*** | NM_004994 | 1.93 | 3.77E-13 |
| ***30*** | ***DEFA1*** | NM_005217 | 1.90 | 3.06E-12 |
| ***31*** | ***SELENBP1*** | NM_003944 | 1.88 | 2.51E-14 |
| ***32*** | ***PGLYRP1*** | NM_005091 | 1.84 | 7.11E-15 |
| ***33*** | ***E4F1*** | NM_004424 | 1.77 | 4.41E-08 |
| ***34*** | ***EPB42*** | NM_001114134 | 1.75 | 5.33E-14 |
| ***35*** | ***KHDRBS2*** | NM_152688 | 1.75 | 1.09E-05 |
| ***36*** | ***CD177*** | NM_020406 | 1.74 | 0.00 |
| ***37*** | ***IFT140*** | NM_014714 | 1.71 | 1.50E-10 |
| ***38*** | ***IFT1B*** | NM_001010987 | 1.69 | 1.28E-10 |
| ***39*** | ***CA4*** | NM_000717 | 1.66 | 7.66E-08 |
| ***40*** | ***CYP4F3*** | NM_001199209 | 1.64 | 0.00 |
| ***41*** | ***P2RX6*** | NM_005446 | 1.61 | 1.85E-04 |
| ***42*** | ***TRIM10*** | NM_006778 | 1.60 | 9.40E-05 |
| ***43*** | ***CA1*** | NM_001128830 | 1.59 | 2.28E-13 |
| ***44*** | ***CTSG*** | NM_001911 | 1.58 | 1.32E-13 |
| ***45*** | ***C4BPA*** | NM_000715 | 1.55 | 7.24E-08 |
| ***46*** | ***SLC4A1*** | NM_000342 | 1.55 | 5.62E-14 |
| ***47*** | ***SOBP*** | NM_018013 | 1.53 | 4.76E-05 |
| ***48*** | ***MPO*** | NM_000250 | 1.50 | 0.00 |
| ***49*** | ***TCTEX1D1*** | NM_152665 | 1.50 | 7.19E-05 |
| ***50*** | ***CYP4F12*** | NM_023944 | 1.494 | 3.65E-07 |
| ***51*** | ***HBA1/HBA2*** | NM_000558 | 1.492 | 2.89E-10 |
| ***52*** | ***HBG1*** | NM_000559 | 1.486 | 1.73E-13 |
| ***53*** | ***CALML6*** | NM_138705 | 1.485 | 9.35E-05 |
| ***54*** | ***MYL4*** | NM_002476 | 1.482 | 0.00E00 |
| ***55*** | ***OLR1*** | NM_002543 | 1.48 | 1.67E-08 |
| ***56*** | ***WDR49*** | NM_178824 | 1.459 | 4.09E-07 |
| ***57*** | ***CRISP3*** | NM_001190986 | 1.455 | 8.57E-12 |
| ***58*** | ***CAMP*** | NM_004345 | 1.454 | 5.57E-14 |
| ***59*** | ***ARHGEF10*** | NM_014629 | 1.417 | 5.74E-13 |
| ***60*** | ***TCN1*** | NM_001062 | 1.41 | 0.00E00 |
| ***61*** | ***HBD*** | NM_000519 | 1.402 | 2.85E-13 |
| ***62*** | ***LCN2*** | NM_005564 | 1.401 | 6.58E-13 |
| ***63*** | ***FOLR3*** | NM_000804 | 1.385 | 2.34E-12 |
| ***64*** | ***AC022007.5*** | NM_001008737 | 1.355 | 1.51E-04 |
| ***65*** | ***HLA-DQA2*** | NM_020056 | 1.339 | 5.27E-12 |
| ***66*** | ***CSMD1*** | NM_033225 | 1.336 | 6.07E-07 |
| ***67*** | ***WASH3P*** | NR_003659 | 1.336 | 7.95E-05 |
| ***68*** | ***AZU1*** | NM_001700 | 1.33 | 0.00E00 |
| ***69*** | ***S100P*** | NM_005980 | 1.315 | 4.42E-12 |
| **70** | ***ADRA2A*** | NM_000681 | 1.301 | 4.26E-06 |
| **71** | ***SCN3A*** | NM_001081676 | 1.284 | 2.23E-06 |
| **72** | ***ADAM23*** | NM_003812 | 1.242 | 3.56E-05 |
| **73** | ***ZNF630*** | NR_033730 | 1.236 | 1.42E-04 |
| **74** | ***SAMD14*** | NM_174920 | 1.232 | 3.20E-13 |
| **75** | ***GIPC3*** | NM_133261 | 1.195 | 1.17E-10 |
| ***76*** | ***RETN*** | NR_029395 | -5.57 | 2.16E-23 |
| ***77*** | ***KIR3DL1*** | NM_002899 | -5.47 | 1.21E-21 |
| ***78*** | ***GFRA2*** | NM_000587 | -4.58 | 5.93E-04 |
| ***80*** | ***OLFML2B*** | NR_003084 | -4.46 | 1.19E-04 |
| ***81*** | ***EHHADH*** | NM_019119 | -4.32 | 2.38E-04 |
| ***82*** | ***IL1B*** | NM_022052 | -4.01 | 6.75E-37 |
| ***83*** | ***CLEC4F*** | NM_032587 | -2.39 | 1.85E-04 |
| ***84*** | ***NRARP*** | NM_005953 | -2.38 | 0.00 |
| ***85*** | ***EFNA5*** | NM_001128209 | -2.31 | 2.42E-15 |
| ***86*** | ***ARHGEF25*** | NR_001447 | -2.26 | 1.55E-07 |
| ***87*** | ***ITGAD*** | NM_024582 | -1.96 | 2.25E-05 |
| ***88*** | ***HRASLS2*** | NM_017878 | -1.94 | 1.24E-10 |
| ***89*** | ***FAT4*** | NM_005353 | -1.84 | 7.16E-06 |
| ***90*** | ***MT1L*** | NM_001111270 | -1.82 | 3.03E-07 |
| ***91*** | ***SGCD*** | NM_001962 | -1.82 | 1.12E-07 |
| ***92*** | ***MT2A*** | NM_001004354 | -1.76 | 2.11E-04 |
| ***93*** | ***CARD6*** | NM_173535 | -1.73 | 7.71E-38 |
| ***94*** | ***NXF3*** | NM_000576 | -1.69 | 2.69E-277 |
| ***95*** | ***PCDHB9*** | NM_001166415 | -1.61 | 1.66E-04 |
| ***96*** | ***HOXC5*** | NM_015441 | -1.55 | 7.26E-05 |
| ***97*** | ***C7*** | NM_001165039 | -1.53 | 2.27E-07 |
| ***98*** | ***RBP1*** | NM_013289 | -1.50 | 1.10E-203 |
| ***99*** | ***IGLL3P*** | NM_020415 | -1.50 | 1.80E-76 |
| **99** | ***BOK*** | NM_032515 | -1.48 | 2.07E-13 |
| **100** | ***FAM161B*** | NM_152445 | -1.47 | 1.23E-04 |
| **101** | ***ITGB4*** | NM_000213 | -1.43 | 2.14E-04 |
| **102** | ***GPR34*** | NM_001097579 | -1.40 | 3.03E-11 |
| **103** | ***MYOM2*** | NM_003970 | -1.37 | 8.14E-169 |
| **104** | ***COLGALT2*** | NM_015101 | -1.36 | 2.78E-14 |
| **105** | ***PDGFRB*** | NM_002609 | -1.35 | 1.50E-46 |
| **106** | ***KIF19*** | NM_153209 | -1.33 | 8.12E-05 |
| **107** | ***SNAI1*** | NM_005985 | -1.32 | 1.97E-08 |
| **108** | ***KLRC4-KLRK1/KLRK1*** | NM_001199805 | -1.30 | 1.74E-05 |
| **109** | ***PODN*** | NM_001199082 | -1.29 | 5.99E-05 |
| **110** | ***PRSS57*** | NM_214710 | -1.26 | 1.87E-26 |
| **111** | ***SMKR1*** | NM_001195243 | -1.24 | 7.64E-09 |
| **112** | ***PPP2R2B*** | NM_001127381 | -1.22 | 1.82E-10 |
| **113** | ***EREG*** | NM_001432 | -1.20 | 1.02E-32 |
| **114** | ***KIR2DL5B*** | NM_001018081 | -1.20 | 8.93E-06 |

**Supporting Table C. The commonly shared identified differently expressed genes between RA *vs.* Control and T2D *vs.* Control**

| **No.** | **Gene symbol** | **ID** | **Log 2 Ratio** | |
| --- | --- | --- | --- | --- |
|  |  |  | **RA *vs.* control** | **T2D *vs.* control** |
| **1** | ***RNF17*** | NM_031277 | 4.81 | 5.70 |
| **2** | ***BMX*** | NM_001721 | 4.05 | 3.07 |
| **3** | ***RNF182*** | NM_001165034 | 3.46 | 5.29 |
| **4** | ***C4BPA*** | NM_000715 | 3.32 | 1.56 |
| **5** | ***BTNL3*** | NM_197975 | 3.00 | 2.52 |
| **6** | ***LOC642236*** | NR_033907 | 2.44 | 2.17 |
| **7** | ***CD177*** | NM_020406 | 2.40 | 1.74 |
| **8** | ***CA4*** | NM_000717 | 2.26 | 1.66 |
| **9** | ***ARG1*** | NM_000045 | 2.22 | 2.01 |
| **10** | ***BPI*** | NM_001725 | 2.06 | 2.39 |
| **11** | ***HBG1*** | NM_000559 | 1.95 | 1.49 |
| **12** | ***MMP9*** | NM_004994 | 1.90 | 1.93 |
| **13** | ***FOLR3*** | NM_000804 | 1.85 | 1.39 |
| **14** | ***DEFA1*** | NM_005217 | 1.85 | 1.90 |
| **15** | ***CYP4F3*** | NM_001199209 | 1.77 | 1.64 |
| **16** | ***LTF*** | NM_001199149 | 1.71 | 2.17 |
| **17** | ***AHSP*** | NM_016633 | 1.65 | 1.93 |
| **18** | ***MYL4*** | NM_002476 | 1.59 | 1.48 |
| **19** | ***S100P*** | NM_005980 | 1.47 | 1.32 |
| **20** | ***HBD*** | NM_000519 | 1.40 | 1.40 |
| **21** | ***HLA-DQA2*** | NM_020056 | 1.31 | 1.34 |
| **22** | ***SELENBP1*** | NM_003944 | 1.26 | 1.88 |
| **23** | ***CA1*** | NM_001128830 | 1.24 | 1.59 |
| **24** | ***MPO*** | NM_000250 | 1.23 | 1.50 |
| **25** | ***DEFA4*** | NM_001925 | 1.22 | 2.03 |
| **26** | ***MMP8*** | NM_002424 | 1.20 | 1.96 |
| **27** | ***CEACAM8*** | NM_001816 | 1.18 | 1.96 |
| **28** | ***RBP1*** | NM_002899 | -7.06 | -5.47 |
| **29** | ***IGLL3P*** | NR_029395 | -4.35 | -5.58 |
| **30** | ***ITGB4*** | NM_000213 | -2.48 | -1.43 |
| **31** | ***SNAI1*** | NM_005985 | -1.67 | -1.37 |
| **32** | ***MT2A*** | NM_005953 | -1.25 | -2.38 |
